# Green Synthesis of Metallic Nanoparticles from *Quercus* Bark Extracts: Characterization and Functional Properties

**DOI:** 10.3390/antiox13070822

**Published:** 2024-07-09

**Authors:** Năstaca-Alina Coman, Alexandra Nicolae-Maranciuc, Lavinia Berța, Alexandru Nicolescu, Mihai Babotă, Adrian Man, Dan Chicea, Lenard Farczadi, László Jakab-Farkas, Barbara Silva, Jéssica Veiga-Matos, Corneliu Tanase

**Affiliations:** 1 Doctoral School of Medicine and Pharmacy, “George Emil Palade” University of Medicine, Pharmacy, Sciences and Technology of Târgu Mures, 38 Gheorghe Marinescu Street, 540139 Târgu Mures, Romania; nastaca-alina.coman@umfst.ro; 2 Research Center for Complex Physical Systems, Faculty of Sciences, Lucian Blaga University of Sibiu, 550012 Sibiu, Romania; alexandra.nicolae@ulbsibiu.ro (A.N.-M.); dan.chicea@ulbsibiu.ro (D.C.); 3 Institute for Interdisciplinary Studies and Research (ISCI), Lucian Blaga University of Sibiu, 550024 Sibiu, Romania; 4 Department of General and Inorganic Chemistry, “George Emil Palade” University of Medicine, Pharmacy, Sciences and Technology of Târgu Mures, 38 Gheorghe Marinescu Street, 540139 Târgu Mures, Romania; lavinia.berta@umfst.ro; 5 Laboratory of Chromatography, Institute of Advanced Horticulture Research of Transylvania, Faculty of Horticulture and Business in Rural Development, University of Agricultural Sciences and Veterinary Medicine, 3–5 Mănăștur Street, 400372 Cluj-Napoca, Romania; babotamihai95@gmail.com; 6 Research Center of Medicinal and Aromatic Plants, “George Emil Palade” University of Medicine, Pharmacy, Sciences and Technology of Târgu Mures, 38 Gheorghe Marinescu Street, 540139 Târgu Mures, Romania; corneliu.tanase@umfst.ro; 7 Department of Microbiology, Faculty of Medicine, “George Emil Palade” University of Medicine, Pharmacy, Sciences and Technology of Târgu Mures, 38 Gheorghe Marinescu Street, 540139 Târgu Mures, Romania; adrian.man@umfst.ro; 8 Chromatography and Mass Spectrometry Laboratory, Center for Advanced Medical and Pharmaceutical Research, “George Emil Palade” University of Medicine, Pharmacy, Sciences and Technology of Târgu Mures, 38 Gheorghe Marinescu Street, 540139 Târgu Mures, Romania; lenard.farczadi@umfst.ro; 9 Faculty of Technical and Human Sciences, Sapientia Hungarian University of Transylvania, 540485 Târgu Mures, Romania; jflaci@ms.sapientia.ro; 10 UCIBIO—Applied Molecular Biosciences Unit, Laboratory of Toxicology, Department of Biological Sciences, Faculty of Pharmacy, University of Porto, Rua de Jorge Viterbo Ferreira n° 228, 4050-313 Porto, Portugal; barbarapolerisilva@gmail.com (B.S.); jessicamatos1996@outlook.com (J.V.-M.); 11 Associate Laboratory i4HB—Institute for Health and Bioeconomy, Faculty of Pharmacy, University of Porto, 4050-313 Porto, Portugal; 12 Department of Pharmaceutical Botany, Faculty of Pharmacy, “George Emil Palade” University of Medicine, Pharmacy, Sciences and Technology of Târgu Mures, 38 Gheorghe Marinescu Street, 540139 Târgu Mures, Romania

**Keywords:** biosynthesis, silver and gold nanoparticles, *Quercus*, rhytidome, phenolic compounds, antioxidant, antimicrobial

## Abstract

*Quercus* species are utilized for their durable wood, providing sustenance for wildlife, conserving biodiversity, and contributing ecological, medicinal, and esthetic benefits to ecosystems and landscapes. In this study, we aimed to use the bark of three *Quercus* species (*Q. dalechampi*, *Q. fraineto*, and *Q. petraea*) for the synthesis of silver and gold nanoparticles (AgNPs and AuNPs). The aqueous extracts from the bark of *Quercus* sp. acted both as reducing and stabilizing agent, facilitating the rapid synthesis of AuNPs (AuQD, AuQF, and AuQP) and AgNPs (AgQD, AgQF, and AgQP). The obtained nanoparticles were characterized using UV-vis spectroscopy, TEM, DLS, and FTIR. Characterizations revealed that the nanoparticles exhibited a variety of shapes, such as polygonal, triangular, and spherical forms, with sizes ranging between 14 and 24 nm for AuNPs and 45–70 nm for AgNPs. The total phenolic content was assessed through spectroscopic methods, while several individual phenolic compounds were identified and quantified using UPLC-PDA. Furthermore, we assessed the antioxidant, antibacterial, and antifungal capacities of AuNPs, AgNPs, and raw extracts. The highest antioxidant activity was observed for raw extracts, followed by AgNPs and AuNPs, while the most potent antibacterial and antifungal activity was observed in AgQP. Moreover, cytotoxicity was examined in a human keratinocyte cell line (HaCaT). The results indicated no cytotoxic effects for AuNPs, while AgNPs and the raw extracts exhibited cytotoxic effects after 48 h of incubation. This research underscores the multifaceted utility of *Quercus* bark extracts in the green synthesis of metallic nanoparticles and their subsequent bioactivity assessment, suggesting promising perspectives for their application in various fields while urging cautious consideration of their cytotoxic implications.

## 1. Introduction

Plant extracts are applied for the synthesis of metal nanoparticles because they offer an environmentally friendly and sustainable approach, thus reducing the use of toxic chemicals [1,2]. Typically, they contain bioactive compounds, such as polyphenols and flavonoids, which present reducing and stabilizing properties, facilitating the formation and stabilization of nanoparticles. This approach is simple, efficient, and economical, providing control over the size and shape of the synthesized nanoparticles while being biocompatible and generating less waste [3]. Recently, various plant derivatives have been used to produce silver and gold nanoparticles (AgNPs and AuNPs) due to their high content in bioactive compounds, which are especially rich in antioxidant species. *Camellia sinensis* tea leaves are preferred for their high content of polyphenols and flavonoids that can assist in reducing different metal ions [4]. *Azadirachta indica* leaf extract contains bioactive compounds such as azadirachtin and quercetin, which facilitate the production of nanoparticles [5]. *Ocimum sanctum* leaf extract is utilized for its content of antioxidant compounds [6], while *Punica granatum* bark is rich in tannins and polyphenols, known as effective redox agents [7]. Moreover, *Mentha piperita* leaf extracts contain flavonoids and polyphenols that contribute to the reduction of metal ions [8].

Certain research studies indicate that when plant extracts are combined with nanoparticles, they can synergize to enhance their effects [9]. This has the potential to amplify the efficiency of nanoparticles in combating pathogenic microorganisms and offering protection against their damage. Researchers have explored the development of metal nanoparticles, such as AgNPs and AuNPs, because of their adaptability in improving pharmaceutical and medical products. The color properties of these nanoparticles are impacted by a range of factors, such as their shape, size, level of clustering, and other attributes like stability, responsiveness, and consistency [10]. The currently developed AgNPs are commonly utilized in varied fields, such as biomedicine, packaging technology, catalysis, detection and elimination of pollutants from water and air, and environmental cleansing [11,12]. Recently, some research studies have even suggested that AuNPs produced with plant extracts have the potential to combat cancer and viruses [13]. In the last few years, in addition to the formation of AuNPs and AgNPs, plant extracts have also been used for the synthesis of other metallic nanoparticles. As illustrative examples, zinc nanoparticles (ZnNPs) were obtained using *Moringa oleifera* [14] and *Aloe vera* leaf extracts [15], copper nanoparticles (CuNPs) were obtained using *Eucalyptus* [16] and *Camellia sinensis* leaf extracts [17], titanium nanoparticles (TiO_2_NPs) were obtained using green tea [18] and *Moringa oleifera* extracts [19], iron nanoparticles (FeNPs) were obtained using *Azadirachta indica* [20] and garlic (*Allium sativum*) leaf extracts [21], and platinum nanoparticles (PtNPs) were obtained using extracts of leaves of *Ocimum sanctum* [22].

It has been shown before that nanoparticles can be quite effective in slowing down the growth of bacteria, including Gram-positive bacteria such as *Staphylococcus aureus* and *Bacillus anthracis*, as well as Gram-negative bacteria, such as *Escherichia coli* and *Pseudomonas aeruginosa* [8,23]. The mechanisms through which AgNPs and AuNPs operate against microbes are not fully understood, although two main theories have been proposed. These nanoparticles are believed to interact directly with the cell membranes of microorganisms, which in turn could destabilize the cell membranes, resulting in the release of cellular content and consequent microbial death [23,24]. In the case of AgNPs, additional effects can be based on the release of silver ions into the environment. These ions can then interact with microorganisms by affecting enzymatic processes or inducing oxidative stress in microbial cells [25]. Nonetheless, the exact mechanism of action of AuNPs remains uncertain [26].

The use of metal nanoparticles in medical applications presents significant risks regarding their toxicity. These particles can cause oxidative stress in cells, leading to apoptosis and necrosis, and trigger inflammatory responses that cause tissue damage. In some cases, they can interact with the DNA molecule, causing genetic damage. Furthermore, nanoparticles can be absorbed and distributed to various organs, including the liver and kidneys, which can lead to systemic toxicity. Therefore, further research is crucial to understand the mechanisms of toxicity and to develop strategies to reduce the associated risks [27,28].

The purpose of this study was the development of silver (AgNPs) and gold (AuNPs) nanoparticles using biologically active natural extracts derived from the bark of certain oak tree species, specifically *Q. dalechampii* (QD), *Q. frainetto* (QF), and *Q. petraea* (QP). Moreover, we aimed to assess their in vitro biological activity and characterize them using UV-vis spectroscopy, TEM, DLS, and FTIR. This approach was chosen to explore the potential of *Quercus* extracts in mediating nanoparticle synthesis as a future research topic, with applicability in the pharmaceutical sector.

## 2. Materials and Methods

### 2.1. Materials

The chemical reagents, such as AgNO_3_ (silver nitrate), HAuCl_4_·3H_2_O (hydrogen tetrachloroaurate(iii) trihydrate), DPPH (2,2-diphenyl-1-picrylhydrazyl), ABTS (2,2′-azino-bis(3-ethylbenzothiazoline-6-sulfonic acid)), CuCl_2_ (copper chloride), FeCl_3_ (ferric chloride), TPTZ (2,4,6-tris(2-pyridyl)-s-triazine), neocuproine, and resazurin were purchased from Sigma-Aldrich in Steinheim, Germany. Folin–Ciocâlteu reagent was obtained from Merck (Darmstadt, Germany).

We used six strains of bacteria: *Staphylococcus aureus* ATCC 25923, Methicillin resistant *Staphylococcus aureus* ATCC 43300, *Enterococcus faecalis* ATCC 29212, *Escherichia coli* ATCC 25922, *Klebsiella pneumoniae* ATCC 13883 and *Pseudomonas aeruginosa* ATCC 27853. Moreover, we used three fungal strains: *Candida albicans* ATCC 10213, *Candida krusei* ATCC 6258, and *Candida auris* ATCC 10913. These microorganisms were sourced from the Department of Microbiology at the University of Medicine, Pharmacy, Sciences, and Technology “George Emil Palade” from Târgu Mureș, Romania.

The materials used for cell culture, including Dulbecco’s Modified Eagle’s Medium (DMEM) with 4.5 g/L of glucose and GlutaMAX™, heat-inactivated treated fetal bovine serum (FBS), 0.25% trypsin/1 mM ethylenediaminetetraacetic acid (EDTA), antibiotics (10,000 units per milliliter of penicillin, 10,000 micrograms per milliliter of streptomycin) and Hanks’ Balanced Salt Solution (HBSS) without calcium and magnesium [HBSS (-/-)] were acquired from GibcoTM (Thermo Fisher Scientific, Alfagene, Portugal). Neutral red (NR) solution and DMSO were obtained from Sigma-Aldrich, Taufkirchen, Germany. All the reagents utilized were either of analytical grade or the highest available quality. The cell line and all the cell culture materials used in this study were obtained from the Faculty of Pharmacy of the University of Porto, Portugal.

### 2.2. Sample Collection and Plant Extracts

The bark was obtained from specimens of *Q. dalechampii* (QD), *Q. frainetto* (QF), and *Q. petraea* (QP) vegetating in the Zagra region of Bistrița Năsăud County, Romania. The species were identified by Professor Dr. Corneliu Tanase from the Department of Pharmaceutical Botany. In the extraction process, the residual bark was used, having an age between 30 and 40 years. The collected bark was then dried in a Nahita 631 drying oven (Auxilab S.L., Beriáin, Spain) at 50 °C and for 24 h. Following dehydration, the dried material was powdered using a Pulverisette 15 cutting mill (Fritsch GmbH, Idar Oberstein, Germany). The resulting biomass was used directly, without any additional processing.

The obtained bark powder was weighed into an Erlenmeyer flask (10 g), mixed with 100 mL of distilled water, and subjected to extraction in an ultrasonic bath [Professional Ultrasonic Cleaner MRC (Beijing, China): AC 150 H, 150 W, 40 kHz, heating power 300 W] for a total of 30 min and at a temperature of 60 °C [29]. The extractive mixtures were vacuum-filtered, and the supernatants were centrifuged for 2 min at 10.000 rpm. Aqueous extracts were used for the nanoparticles’ synthesis, while for the bioactive potential and chemical profile assessments, extracts were freeze-dried and stored in a cool and dry environment until the analysis.

### 2.3. Biosynthesis of Gold Nanoparticles and Silver Nanoparticles

The QD, QF, and QP extracts obtained as mentioned above were utilized to obtain three solutions of gold nanoparticles (AuNPs) and three solutions of silver nanoparticles (AgNPs). For AgNPs, 225 mL of each extract was combined with 25 mL of a 1 mM AgNO_3_ solution, with adjustment to pH = 9.0 by treatment with 1 M NaOH solution. The synthesis process for AgNPs occurred in a bath at a temperature of 60 °C for 3 h, leading to a color transformation from light brown to dark brown. On the other hand, for the synthesis of AuNPs, 225 mL of each extract was mixed with 25 mL of a 1 mM HAuCl_4_ solution at a pH = 3.5. The reduction of Au^3+^ ions was visually confirmed by a change in color from brown to dark reddish purple within just a couple of minutes (1–2 min). The reduction process for HAuCl_4_ ions took place at 35 °C in a bath and lasted between 20 and 25 min.

After nanoparticle synthesis, they were dried through lyophilization using a BK FD12S freeze dryer (Biobase Biodustry Co., Ltd., in Jinan, China). Six types of nanoparticles were obtained: AuQD—*Q. dalechampii* rhytidome, AuQF—*Q. frainetto* rhytidome, AuQP—*Q. petraea* rhytidome, AgQD—*Q. dalechampii* rhytidome, AgQF—*Q. frainetto* rhytidome, and AgQP—*Q. petraea* rhytidome.

### 2.4. Identification and Quantification of Phenolic Compounds by High-Performance Liquid Chromatography (HPLC)

Prior to the identification of the phenolic compounds, each extract was subjected to centrifugation and filtration through a 0.2 µm nylon membrane before injection. Chemical composition analysis of QD, QF, and QP bark extracts before and after biosynthesis of AuNPs and AgNPs was performed by high-performance liquid chromatography (HPLC), including the quantification of specific compounds. The method was based on the one described by Tanase et al. [30], with separation conducted on a Luna C18 column (particle size 3 µm, dimensions 150 mm × 4.6 mm) at a flow rate of 1 mL/min. The HPLC system was coupled to a PDA detector. The elution gradient consisted of 0.1% formic acid in water solution (phase A) and acetonitrile (phase B), transitioning from 90% A/10% B to ratios, over specified time intervals. A thorough examination took place at five preferential wavelengths (270, 280, 324, and 370 nm, respectively), using the standards catechin, epicatechin, taxifolin, chlorogenic acid, sinapic acid, luteolin-7-O-glucoside, gallic acid, quercetin, caffeic acid, vanillic acid, rutin, ellagic acid, eleutheroside B, and quercetin. Each standard solution was injected in a volume of 20 µL.

### 2.5. Characterization of Synthesized AuNPs and AgNPs

#### 2.5.1. UV-VIS

UV–visible spectroscopy (UV-vis) was the method used for monitoring the development of metal nanoparticles. The UV-vis spectrum was recorded using a Specord 210 Plus190 spectrometer (Analytik Jena, Jena, Germany). Both AuNPs and AgNPs were scanned in a 1 cm quartz cuvette within the absorbance range of 350–700 nm while using water as a reference.

#### 2.5.2. Fourier Infrared Spectroscopy (FT-IR)

The FT-IR spectroscopy was performed to detect the chemical modifications that appear in the synthesis process of AgNPs and AuNPs, using the proposed extracts and to confirm their interactions with the extracts once the nanoparticles were formed. After examining the functional groups identified in the samples, valuable chemical information about nanoparticle synthesis was obtained. This characterization was performed using an FT-IR ALPHA spectrophotometer with an ATR crystal (Bruker, Billerica, MA, USA). The analysis was realized on the lyophilized samples based on 24 scan times at a resolution of 4 cm^−1^ with a wavelength in the range of 400–4000 cm^−1^ and their absorbance values were recorded. For a complete overview of the samples’ insights, FT-IR was performed for extracts as controls and for the nanoparticles based on silver and gold with the specific extract.

#### 2.5.3. TEM and DLS

The AuQD, AuQF, AuQP, AgQD, AgQF, and AgQP that were obtained underwent an analysis of their structure using a transmission electron microscope (TEM). The Hitachi H 7650 automated transmission electron microscope operates at 120 kV on a copper grid coated with carbon. The size of the synthesized AuNPs and AgNPs was determined using dynamic light scattering (DLS). In this method, a coherent light beam is directed towards particles suspended in a solvent to produce a scattered light pattern. The intensity of interference is captured by a detector, and the data acquisition system (DAS) records it as a time series. This technique was adapted from work by Chicea et al. [31]. Error analysis was carried out accordingly. It was observed that there is a 13% error in measuring particle diameters. Despite this margin of error being notable, it aligns with the strategy of streamlining the setup and data processing steps for DLS procedures.

### 2.6. Total Phenolic Content

The technique for assessing the total polyphenolic content (TPC) of the extracts was outlined in a previous study by Mocan et al. [32]. To summarize, a mixture of Folin–Ciocalteu reagent (1:10 *v/v*, in water) and samples were prepared in a 96-well plate. Following an incubation at room temperature, a sodium carbonate solution (7.5% *w*/*v*) was introduced to each well, followed by another incubation period. After 30 min, the absorbance of the mixture was measured at 760 nm using a SPECTROstar Nano microplate reader (BMG Labtech, Offenburg, Germany). The TPC findings for both extracts and nanoparticles were presented as milligrams of gallic acid equivalents per gram of the lyophilized extracts or nanoparticles (mg GAE/g dw).

### 2.7. In Vitro Antioxidant Activity Assessment

The levels of antioxidants in AuQD, AuQF, AuQP, AgQD, AgQF, AgQP, and the extracts (QD, QF, and QP) were assessed through four in vitro tests: DPPH, ABTS, FRAP, and CUPRAC, using a SPECTROstar Nano Multi Detection microplate reader with 96-well plates (BMG Labtech, in Ortenberg, Germany). All samples were tested at a concentration of 1 mg/mL in 50% *v/v* ethanol supplemented with 5% DMSO, and the solvent mix was used as the detection blank for all these assays. The results were expressed as Trolox equivalents per gram of lyophilized extracts or nanoparticles (mg TE/g dw).

#### 2.7.1. DPPH Assay

The DPPH radical scavenging ability was assessed using the procedure outlined by Babotă et al. [33]. Reaction mixtures (30 μL of sample solution and 270 μL of 0.002% DPPH methanolic solution) were pipetted in a 96-well plate, incubated for 30 min in the dark, and subsequently read at 570 nm.

#### 2.7.2. FRAP Assay

During the FRAP assay, the conversion of Fe^3+^-TPTZ to the Fe^2+^-TPTZ complex shows a change in coloration. Briefly, 175 μL of FRAP reagent and 25 μL of the sample solution were pipetted in a 96-well plate. This mixture was then left to incubate for half an hour at room temperature, following which the final absorbance at 593 nm was measured [34].

#### 2.7.3. ABTS Assay

The ABTS radical scavenging ability was assessed using the procedure outlined by Babotă et al. [33]. Equal volumes of 7 mM ABTS^+^ with 2.45 mM K_2_S_2_O_8_ aqueous solutions were mixed to obtain a radical stock solution overnight and then further diluted to a maximum absorption of 900 at maximal wavelength. Next, 200 μL of radical solution was placed in a 96-well plate with 20 μL of each sample and incubated for 6 min before reading the absorbance at 734 nm.

#### 2.7.4. CUPRAC Assay

The CUPRAC test followed the procedure outlined by Mustafa et al. [35], with adjustments. Each sample (25 μL) was combined with 175 μL of CUPRAC reagent (equal volumes of 0.1 mM CuCl_2_ solution, 0.075 nM neocuproine in ethanol, and ammonium acetate buffer, with pH = 7.0). The absorbance was measured at 450 nm after 30 min of incubation.

### 2.8. Antibacterial Activity

The effectiveness of AuNPs, AgNPs, and extracts against a group of three Gram-positive bacteria (*Staphylococcus aureus*, Methicillin-resistant *Staphylococcus aureus*, and *Enterococcus faecalis*) and three Gram-negative bacteria (*Escherichia coli*, *Klebsiella pneumoniae*, and *Pseudomonas aeruginosa*) was examined. A microdilution method, as described previously, was used to assess their antimicrobial activity directly on 96-well microplates [36]. Following the preparation of nanoparticle and extract solutions (in 5% DMSO), a range of dilutions were created in water by transferring 100 μL from the column to subsequent columns. Concurrently, a 0.5 McFarland bacterial suspension was made using saline solution and fresh human pathogenic bacterial cultures. A mixture of 10 μL from this suspension with 9990 μL of Mueller Hinton broth 2X (MHB) was then added to each dilution in the plate columns containing nanoparticles and extracts. Furthermore, a control group with water and MHB 2X was added to verify the cleanliness of the culture medium. The microplates were incubated for 24 h at 37 °C. The MIC was detected following the addition of 20 μL of resazurin solution to each well, and the plates were incubated for 2 h at 37 °C. The well where the resazurin retained its color was noted as the Minimum Inhibitory Concentration (MIC). Before adding the 0.015% resazurin, wells with no growth were identified, and 3 μL from these wells was transferred onto a Sheep Blood Agar culture medium (Oxoid Ltd., Hampshire, UK). The plates were then placed in an incubator at 35 °C for 18 h. The spot with no growth on the Sheep Blood Agar plate was considered the Minimum Bactericidal Concentration (MBC).

### 2.9. Antifungal Activity

The antifungal activity of the AuNPs, AgNPs, and extracts was tested against three different strains: *Candida albicans* ATCC 10213, *Candida krusei* ATCC 6258, and *Candida auris* ATCC 10913. The antifungal activity was examined using the microdilution method, as previously described [36]. To summarize, fungal cultures were mixed to a turbidity level of 0.5 on the McFarland scale. Then, they were combined with 9 mL of RPMI medium that was buffered with MOPS and contained 2% glucose. Following this, 100 μL of cultures were exposed to varying dilutions of each NP solution and extract to determine the minimum inhibitory concentration (MIC) values. The Minimum Fungicidal Concentration (MFC) was determined using Sabouraud Agar medium.

### 2.10. Cytotoxic Assays

#### 2.10.1. HaCaT Cell Culture

HaCaT cells, an immortalized human keratinocyte cell line, were obtained from the American Type Culture Collection (ATCC; Manassas, VA, USA) and routinely cultured in 75 cm^2^ flasks. The growth medium consisted of DMEM with 4.5 g/L of glucose and GlutaMAX™, supplemented with 10% heat-inactivated FBS, 100 U/mL of penicillin, and 100 μg/mL of streptomycin. The cells were maintained at a temperature of 37 °C in an environment containing 5% CO_2_ and 95% air, with the medium being changed every 2 to 3 days. Regular trypsinization (0.25% trypsin/1 mM EDTA) was used for the passage of the cultures.

#### 2.10.2. Neutral Red Uptake Assay

The cytotoxicity of the obtained extracts and nanoparticles was assessed using the neutral red (NR) uptake assay, which estimates the living cells by their ability to accumulate the NR dye inside their lysosomes. The cells were seeded in 96-well plates with a density of 60,000 cells/cm^2^. Twenty four hours after seeding, the medium was aspirated, and the cells were treated with the different concentrations of freshly prepared tested compounds.

After 24 h of exposure, the cell culture medium was replaced with fresh medium containing 50 μg/mL of NR. The cells were then incubated in an environment with 5% CO_2_ and 95% air at 37 °C for 40 min. Following this incubation time, the medium was removed, followed by the extraction of the dye absorbed by cells using a mixture of absolute ethyl alcohol and distilled water (in a 1:1 ratio) with acetic acid (5%, *v/v*). The absorbance was later measured at 540 nm using a well plate reader (PowerWaveX BioTek Instruments, Winooski, VT, USA). We assessed toxicity by comparing the percentage of NR uptake relative to control cells (0 μg/mL). The results were obtained from five independent experiments, performed in triplicate. Additionally, Triton™ X 100 (at a concentration of 1%, *v/v*) was used as a positive control.

### 2.11. Statistical Analysis

Statistical analysis was applied using GraphPad Prism 8 (GraphPad Software, San Diego, CA, USA). The significance level was set at α = 0.05 before conducting the assessment. The normality of the data was checked using the Shapiro–Wilk test. The assessment of the antioxidant effect was carried out using one-way ANOVA, with Tukey’s post hoc test, and cytotoxicity assessments were carried out using two-way ANOVA, with Sidak’s post hoc test. For TPC analysis, the Kruskal–Wallis test was used, followed by Dunn’s post hoc test. Any *p*-value < 0.05 was deemed significant. Spearman coefficients were computed to evaluate the relationship between TPC and antioxidant capacity.

## 3. Results and Discussion

### 3.1. Production of AgNPs and AuNPs

One of the most common approaches for the production of AgNPs involves reducing the silver ions in AgNO_3_ in a water-based solution. Past research has highlighted that the color change serves as an indication of nanoparticle formation [37]. As the reduction process takes place, the solution changes from a light brown shade to a dark brown shade, indicating the generation of AgNPs. This transformation occurs as the Ag^+^ ions in the AgNO_3_ solution are converted into metallic silver (Ag^0^) [38,39]. Similarly, in the case of Au nanoparticles, Au^3+^ ions present in HAuCl_4_·3H_2_O are reduced to metallic gold (Au^0^) during the synthesis. In this process, Au^3+^ ions receive electrons and transition into gold [40]. Previous studies have observed color changes to brown and dark violet for Ag and Au nanoparticles, respectively, confirming their successful formation [10,26,41].

### 3.2. HPLC Analysis

Polyphenols are considered a structurally diverse group of phytochemicals found in different species of the *Quercus* genus. Among these, gallic acid, chlorogenic acid, and vanillic acid have been cited in the literature as the most abundant in various species (i.e., *Q. robur*, *Q. petraea*, *Q. phillyraeoides*, *Q. acuta*, and *Q. myrsinaefolia*) [42]. Our investigation primarily focused on examining the presence and content in phenolic compounds in extracts from QD, QF, and QP, as well as in the AuNPs and AgNPs produced using these extracts. The phenolic compounds identified are listed based on their retention times in Table 1. Almost all 13 compounds analyzed were detected in extracts at varying levels, except for vanillic acid. Specifically, sinapic acid was not found in *Q. frainetto,* and caffeic acid, along with luteolin-glucoside, was not detected in *Q. petraea*. The compound with the highest amount found in the extracts was ellagic acid, with a maximal value of 8077.98 μg/mL for the QF extract. However, this phenolic acid was absent in nanoparticle variants (except for AuQD at a concentration of 14.88 μg/mL). This compound in particular is renowned for its anti-inflammatory properties and is currently under research for its possible role in preventing and treating various health issues [43]. Ellagic acid is also acknowledged for its antitumor properties and is being explored in the fields of cancer prevention and supportive therapy [44]. Among the samples that were examined, luteolin-7-O-glucoside was detected in QD and QF. Vanillic acid was detected in QD, QP, and AgQP, whereas sinapic acid was present in QD and QP.

The HPLC analysis also confirms the reduction in Ag^+^ and Au^3+^ ions during the synthesis process, which is indicated by the small concentrations of phenolic constituents in the freeze-dried nanoparticle suspensions. Practically, the obtained results indicated several differences regarding the link between each individual chemical constituent of the extract and its ability to reduce metallic ions. Phenolic acids (such as caffeic, vanillic, ellagic, and sinapic acids) seemed to act as the most potent reducers in comparison with quercetin, epicatechin, or taxifolin, which could still be quantified in several freeze-dried nanoparticle suspensions.

### 3.3. Characterization of AgNPs and AuNPs

The unique physical and chemical properties of nanoparticles give them captivating qualities. To fully understand their nature, it is necessary to examine certain features, such as size, shape, and chemical composition. This knowledge is crucial in customizing nanoparticles for various applications and for understanding their potential effects on the environment and human health. Typically, methods like UV-vis spectroscopy, FTIR spectroscopy, light scattering (DLS) analysis, and transmission electron microscopy (TEM) are utilized to gather details about different nanoparticles.

#### 3.3.1. UV–Visible Absorption Spectroscopy

**Figure 1:**
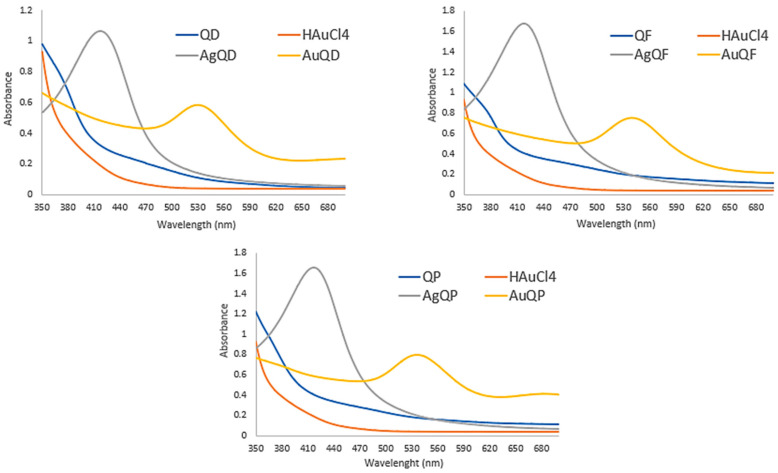
UV–Vis spectrophotometry of AuNP, AgNP, and extract (QD, QF, and QP).

UV–visible absorption spectroscopy is widely utilized for the examination of nanoparticle formation [45]. Figure 1 illustrates distinct variations in the absorption peaks between AuNPs and AgNPs, across different wavelengths. These differences could be attributed to the effects characterized by synchronized oscillations of electrons on the nanoparticles’ surface. The specific wavelength exhibiting this phenomenon is contingent upon different factors, such as nanoparticle size, shape, composition, and dispersal medium [40].

AuNPs showed different absorption peaks, depending on the considered extract. For example, AuQD peaked at 530 nm, AuQF at 540 nm, and AuQP at 536 nm. In contrast, AgQD, AgQF, and AgQP presented identical absorption peaks at 417 nm. As for HAuCl_4_, AgNO_3_, QD, QF, and QP, they did not show absorption peaks. These findings coincide with previous research on both AuNPs [40] and AgNPs [46].

#### 3.3.2. Fourier Infrared Spectroscopy of Green-Synthesized AgNPs and AuNPs

**Figure 2:**
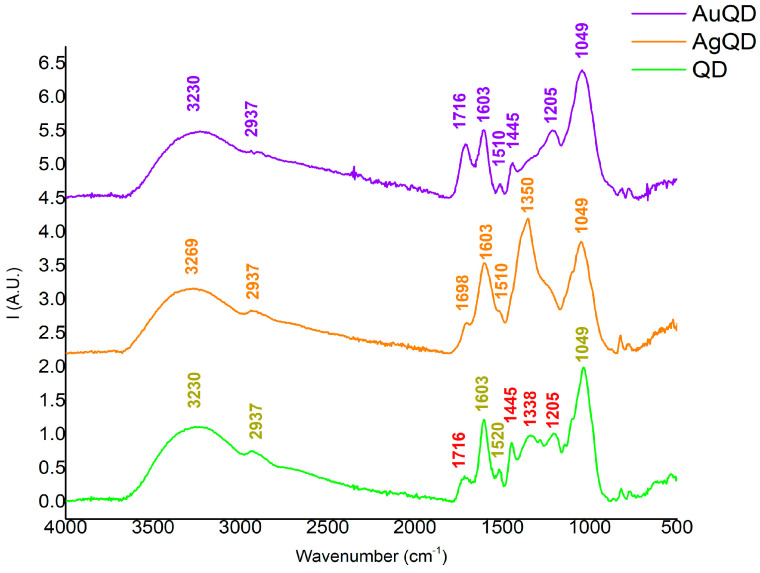
ATR-FTIR spectra of NPs using QD as extract: QD extract (green line), AgNPs based on QD (orange line), and AuNPs based on QD (purple line). The bands marked with red are representative to the ones involved in NPs’ synthesis according to the description.

**Figure 3:**
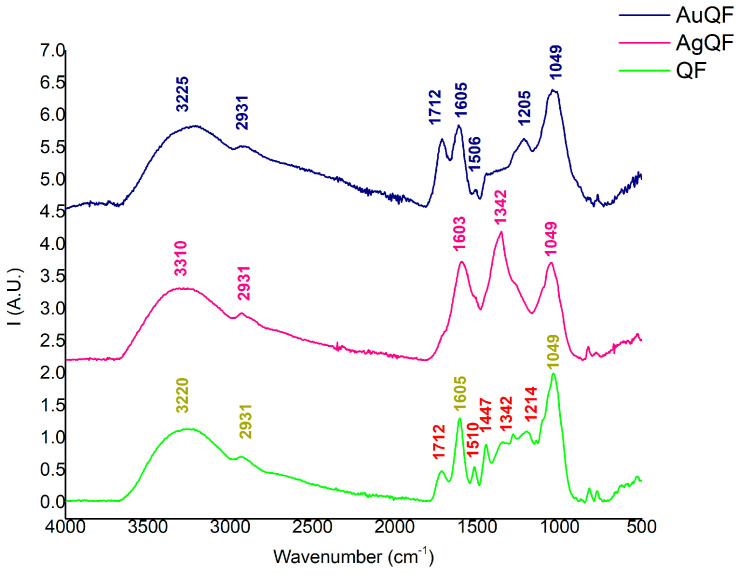
ATR-FTIR spectra of NPs using QF as extract: QF extract (green line), AgNPs based on QF (pink line), and AuNPs based on QF (dark blue line). The bands marked with red are the ones involved in NPs’ synthesis according to the description.

**Figure 4:**
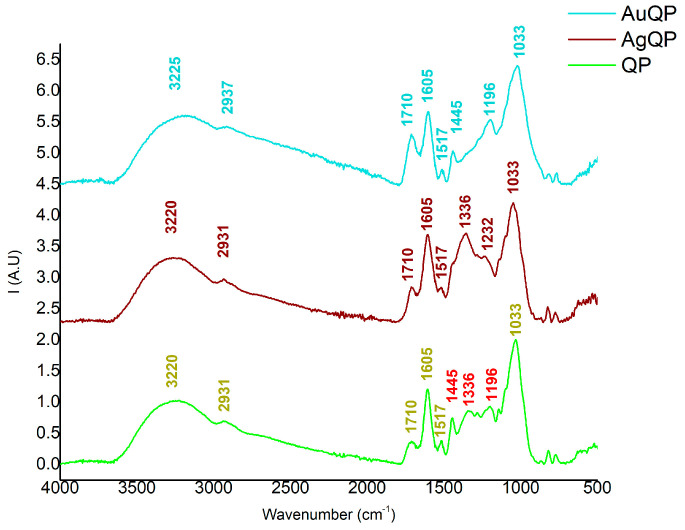
ATR-FTIR spectra of NPs using QP as extract: QP extract (green line), AgNPs based on QP (brown line), and AuNPs based on QP (blue line). The bands marked with red are the ones involved in NPs’ synthesis according to the description.

The FT-IR analysis revealed various chemical groups once the nanoparticles’ syntheses were completed. For a better comparison between the extracts and a better identification of the peaks, the FT-IR spectra are presented separately for each extract. Therefore, the first spectrum is presented in Figure 2, which contains information about both obtained NPs, using QD as an extract. Consequently, the second spectrum regarding AuNPs and AgNPs obtained using QF is presented in Figure 3, while the last spectrum regarding AuNPs and AgNPs obtained using QP is presented in Figure 4.

According to Figure 2, Figure 3 and Figure 4, the AuNPs and AgNPs synthesized proved to have several similar chemical groups, in the case of all three spectra, since similar peaks were observed. The large peaks identified between 3500 and 3200 cm^−1^, with a maximum of 3230 cm^−1^ for the AuNPs and 3269 cm^−1^ for AgNPs using QD, 3220 cm^−1^ for the AgNPs and 3310 cm^−1^ for AuNPs using QF, and 3271 cm^−1^ for the AgNPs, and 3279 cm^−1^ for AuNPs using QP, are associated with vibrational O-H groups [31,47,48] from the organic phase of the extracts. Since all three extracts are rich in polyphenolic structures [49], the presence of O-H bonding reveals that both AuNPs and AgNPs are bound to different components originating in the *Quercus* extracts. The interaction of the organic extract with the NPs generated is also sustained by the appearance of the peak at 2937 cm^−1^ for the NPs synthesized with QD, 2931 cm^−1^ for both the NPs synthesized with QF, and 2937 cm^−1^ for the NPs synthesized using QP. These peaks, which were observed in all three spectra, together with the large region of 3200–3000 cm^−1^, are correlated to asymmetric and symmetric vibrations of C-H-, CH_2_-, and CH_3_-groups from polysaccharides [49,50], a class of metabolites found in large amounts in *Quercus* extracts.

However, for the next regions of the FT-IR spectra, some particularities were identified between the extracts; therefore, the identification of the peaks will be performed individually for each figure. For the QD extract and AuQD, with the spectra displayed in Figure 2, the absorption band appearing at 1716 cm^−1^ is correlated to C=O stretching, a group that can be found in compounds like taxifolin and quercetin. However, in the case of AgQD, there was a small shift to 1698 cm^−1^, which could suggest a possible interaction of these compounds with Ag^2+^ and AgNP formation. At 1603 cm^−1^, the absorbance band indicates the presence of a primary amine, while the signal obtained around 1510 cm^−1^ can be correlated to C=C aromatic ring stretch [51]. Since this last peak also appears in both NPs’ spectra, where the metal precursors themselves do not contain any aromatic compounds, the interaction of extract with metal NPs is strongly suggested, since the extracts are abundant in different aromatic compounds. Another indicator of NPs formation is correlated to the 1445 cm^−1^ peak from QD absorption, which is absent in the AgQD sample. This information suggests that it is highly likely that some organic compounds from the extract can reduce Ag^2+^ ions to Ag^0^ and bind to the AgNPs’ surface, being capped. The peak from 1338 cm^−1^, corresponding to aromatic nitro compounds [52], is reduced for the AuQD compared to the QD spectrum, so a bond is indicated through this structure. Another reduction is observed in the case of AgQD samples where the peak from 1205 cm^−1^ of QD corresponding to C-O stretching vibration is absent. The last peak observed, at 1049 cm^−1^, is attributed to C-C and C-H ring vibrations, since the extract used contains this type of cyclic molecules in a high amount or to C-OH stretching, due to secondary alcohols [53]. Its presence in all samples, including the ones with NPs, suggests a high amount of cyclic compounds in all samples and confirms the suggestion mentioned earlier related to the interaction obtained during the synthesis. Therefore, all the differences observed between QD and AgQD/AuQD spectra prove that many interactions were created between the plant extract and the metal particles during their syntheses.

In the case of the QF extract, with the spectrum displayed in Figure 3, there are similar absorbance bonds comparing AuNPs and AgNPs obtained with QD. At 1712 cm^−1^, the absorbance associated with C=O stretching indicates the first sign of AgQF formation since this peak is reduced, as in the previous reducing agent used. The absence of a C=O chemical group for this sample can appear due to the reduction in quercetin, gallic acid, or ellagic acid. This degradation can occur during the synthesis, and similar observations were highlighted during the HPLC analysis. As in the case of QD extract, the absorbance band at 1605 cm^−1^ indicates the presence of a primary amine, while the signal obtained at around 1506 cm^−1^ can be correlated to the presence of aromatic compounds. Their presence in the samples within the spectra of NPs and extracts indicates the interactions that appear between them in AuNPs and AgNPs formation.

The peak identified at 1447 cm^−1^, associated with C-C stretch from aromatic compounds [54], is absent for both AgQF and AuQD, which confirms that metals can be reduced and nanoparticles can be synthesized through the aromatic compounds present in the extracts. This observation is similar to the case of the QD extract and it is sustained as well by the absence of a 1342 cm^−1^ peak in the case of AuQF (compared to QF), since aromatic nitro compounds are involved, as also mentioned in another study [35]. In the case of AuQF, it can be suggested that a high number of particles bond to these types of compounds due to the high intensity of the peak observed in the spectrum. For the other type of nanoparticles, namely AgQF, their synthesis is strongly suggested by the decrease in many peaks, including the one from 1214 cm^−1^ corresponding to C-O stretching vibration, which is nonetheless present in QF [55].

For the samples produced using QP as extracts, with the spectra presented in Figure 4, fewer reduction peaks were observed in comparison to QD and QF. For example, the peak at 1710 cm^−1^ corresponds to C=O stretching, and the one at 1605 cm^−1^ indicates the presence of a primary amine, while the peak at around 1517 cm^−1^ can be associated with C=C due to aromatic symmetrical stretching. Two reductions were noticed: the peak from 1445 cm^−1^ was reduced in the case of AgQP, while the peak from 1336 cm^−1^ was reduced for AuQP. The absence of the 1336 cm^−1^ band can be associated with the reduction processes of aromatic compounds, since the peak is associated in other studies with C-N stretching vibrations of aromatic amines [35]. Considering the fact that the *Quercus* extracts present elevated levels of phenolic and other aromatic compounds, the interaction of gold ions with an aromatic amine could lead to their reduction and AuNPs’ formation. The last peak from 1033 cm^−1^, associated with cyclic molecules and C-OH stretching, as in the case of previous extracts, is another confirmation of metal–extract interactions [56].

All the presented FT-IR spectra suggest the interactions of the precursors with the reducing agents based on *Quercus extracts* through the identification of reducing peaks. During this analysis, it was proven that QD and QF have a stronger impact on the reducing synthesis compared to QP extract, where only a peak reduction was observed for each ion. Furthermore, AgNPs proved to be more sensitive to *Quercus* extracts, which is sustained by the absence of different chemical groups compared to the extracts used as reference for reduction. For a better visualization, all the chemical vibrations identified are presented in Table 2.

#### 3.3.3. TEM and DLS analyses of AgNPs and AuNPs

**Figure 5:**
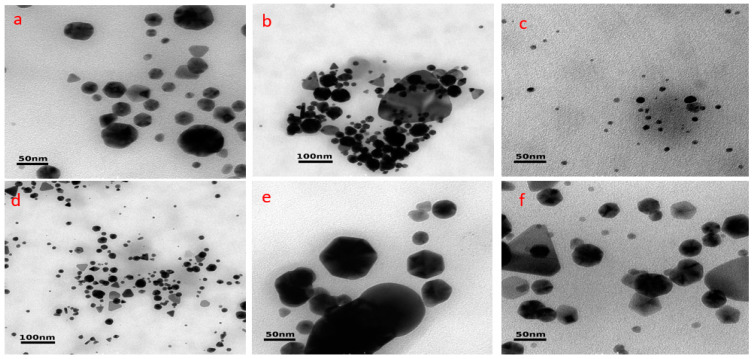
Transmission electron microscope (TEM) images of silver and gold nanoparticles: (**a**) AgQD-silver nanoparticles obtained via *Q. dalechampii* rhytidome; (**b**) AgQF-silver nanoparticles obtained via *Q. frainetto* rhytidome; (**c**) AgQP-silver nanoparticles obtained via *Q. petraea* rhytidome; (**d**) AuQD-gold nanoparticles obtained via *Q. dalechampii* rhytidome; (**e**) AuQF-gold nanoparticles obtained via *Q. frainetto* rhytidome; (**f**) AuQP-gold nanoparticles obtained via *Q. petraea* rhytidome.

TEM was the technique used for the determination of the shape of the nanoparticles, as shown in Figure 5. The AuQD, AuQF, and AuQP had a variety of shapes, including polygonal, triangular, and spherical forms, with sizes of 27.0 ± 3.5 nm for AuQD, 14.4 ± 1.9 nm for AuQF, and 24.1 ± 3.1 nm for AuQP. On the other hand, AgQD and AgQF particles displayed shapes with average diameters of 65.8 ± 8.5 nm and 45.4 ± 5.9 nm, respectively, while most AgQP particles are spherical with an average diameter of 66 ± 8.6 nm. The results align with findings from UV-vis spectroscopy, indicating that smaller AuNPs are favored over AgNPs due to composition and dispersion medium considerations.

Currently, it is crucial to acknowledge the fact that the mean diameter is not simply an arithmetic average of individual values, given that the intensity of light scattering is proportional to the 6th power of the diameter. The interference pattern is predominantly shaped by the presence of the largest particles in the suspension. In scenarios with nearly monodisperse particles, the diameter obtained through dynamic light scattering (DLS) essentially represents the average hydrodynamic diameter, which is slightly different from the physical diameter [57]. Moreover, the DLS technique is not sensitive to the nature of the particles; therefore, the average diameter that is the output of the DLS procedure is the hydrodynamic diameter of all the particles in the suspension, organic or inorganic.

### 3.4. Total Phenolic Content Estimation

**Figure 6:**
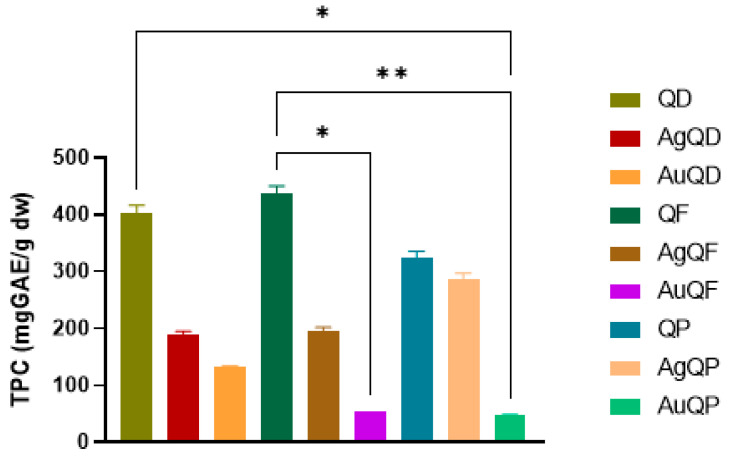
Estimation of total phenolic content based on the Folin–Ciocalteau method. Statistical analysis was performed by Kruskal–Wallis, with Dunn’s multiple comparisons post-test. All determinations were made in triplicate. Data were considered to be statistically significant where ** *p* < 0.01 and * *p* < 0.05.

As depicted in Figure 6, the tested extracts exhibited a value of total phenolic species exceeding 300 mg GAE/g dw, while the TPC levels decreased in the nanoparticles. This decrease can be attributed to the role of phenolics in reducing gold and silver ions, facilitating nanoparticle creation and stability. Consequently, a significant disparity (*p* < 0.05) in content was observed between AuQF and QF extract. Furthermore, notable distinctions were identified between QD and AuQP, as well as between QF and AuQP.

Several research studies have noticed differences in the amounts of phenolic species found in the bark of oak species. For instance, when looking at *Q. crassifolia,* researchers noted that the aqueous bark extract contained a higher content (860 mg GAE/g) in comparison to the ethanolic extract (695 mg GAE/g) [58]. From the studies conducted, it was observed that the aqueous extract of *Q. laurina* bark showed a phenolic content of 474 mg GAE/g, while the ethanolic extract had a higher phenolic content, measured at 756 mg GAE/g. Similarly, the aqueous extract of *Q. scytophylla* bark had a phenolic content of 329 mg GAE/g, while the ethanolic extract showed higher levels of phenolic derivatives, with 521 mg GAE/g [59]. Furthermore, the methanolic extracts from *Q. robur, Q. petraea*, and *Q. pyrenaica* displayed a varied range of concentrations of phenolic compounds. Specifically, values of 72.63 μg GAE/g, 48.87 μg GAE/g, and 41.48 μg GAE/g were found for these species [60]. It is worth noting that variations in quantities among extracts should be noticed considering the use of various solvent systems, extraction methods, and analytical approaches. Moreover, the difference between the tested extracts and nanoparticles could be based on their behavior regarding solubility since the assessment method is applied in an aqueous medium.

### 3.5. Antioxidant Potential of the Nanoparticles and the Extracts

In this study, we have also assessed the in vitro antioxidant potential of nanoparticles and extracts, using four methods, and the results are outlined in Table 3.

Among the samples, the QD extract showed high potential in scavenging DPPH radicals, as well as reducing trivalent iron (Fe^3+^) to divalent iron (Fe^2+^) (during the FRAP assay). Interestingly, gold (AuQD) and silver (AgQD) nanoparticles produced using this QD extract did not exhibit analogous levels of antioxidant activity. This could be due to several factors, one of the most important being the characteristic composition of the nanoparticles, as well as the assessment methods used for antioxidant activity [61]. Notably, AgQP solution showed elevated antioxidant activity using the DPPH, ABTS, FRAP, and CUPRAC tests. This effectiveness is thought to be linked to the presence of extracted phytochemicals, such as phenolic acids and flavonoids, which act as protective agents that can attach to AgNP surfaces and effectively combat free radicals [62].

The study did not determine any connection between the level of polyphenols and their antioxidant properties, suggesting that additional plant compounds present in the samples influenced their antioxidant capabilities. Furthermore, in vitro assays do not possess the ability to perform full chemical profiling and can analyze only specific subsets of antioxidant metabolites.

Silver nanoparticles crafted from the extract of *Elaeagnus umbellata* fruit have displayed an antioxidant capacity of 69% when measured at a concentration of 100 μg/mL. At lower concentrations, such as 50 μg/mL, 25 μg/mL, and 12.5 μg/mL, their antioxidant potential was observed to be moderately effective, at 57.8%, 41.8%, and 37.30%, respectively [63]. The level of antioxidant properties in nanoparticles created using *Moringa oleifera* fruits is affected by both the extract concentration and the length of the incubation period. Silver nanoparticles showed the ability to neutralize DPPH radicals, achieving a rate of 78.37 ± 2.4% at 100 μg/mL and 88.6 ± 3.7% at 200 μg/mL after a half-hour period of incubation. Following in effectiveness were copper oxide nanoparticles and iron nanoparticles, in this order. When compared to BHT, the used standard silver nanoparticles exhibited the highest antioxidant potency against DPPH radicals, with rates of 89.4 ± 1.4% at 100 μg/mL and 92.8 ± 3.2% at the higher concentration of 200 μg/mL [64]. Gold nanoparticles (AuNPs) have shown antioxidant properties while maintaining low toxicity to cells [65].

### 3.6. Application of the Synthesized Silver and Gold Nanoparticles Antibacterial Activity

Table 4 shows the efficacy of AuNPs, AgNPs, QDs, QFs, and QPs against Gram-negative and Gram-positive bacteria. Among all the solutions tested on Gram-positive bacteria (*S. aureus* ATCC 25923, MRSA ATCC 43300, *E. faecalis* ATCC 29212), the results showed that AgQP had the lowest minimum inhibitory concentration (MIC) for *S. aureus* ATCC 25923 (0.31 mg/mL), while QP was effective for MRSA ATCC 43300 (0.31 mg/mL) and AgQP for *E. faecalis* ATCC 29212 (1.25 mg/mL). The minimum bactericidal concentration (MBC) was also studied for Gram-positive bacteria, with remarkable results: AgQP had the lowest MBC for *S. aureus* ATCC 25923 (1.25 mg/mL), AgQF for MRSA ATCC 43300 (1.25 mg/mL), and AgQF and AgQP for *E. faecalis* ATCC 29212 (2.50 mg/mL). In relation to Gram-negative bacteria, the antibacterial activity was lower than in the case of Gram-positive bacteria. Thus, among all the analyzed solutions, the study showed that AgQP had an inhibitory and bactericidal effect against *S. aureus* ATCC 25923 at a concentration of 1.25 mg/mL (MIC = MBC), while QP had an inhibitory effect at 0.31 mg/mL and a bactericidal effect at 0.62 mg/mL for MRSA ATCC 43300. For *E. faecalis* ATCC 29212, both products had an inhibitory effect at 0.62 mg/mL, but only QP had a bactericidal effect at 2.50 mg/mL, while AgQP did at 1.25 mg/mL.

The antibacterial activity of these bacteria was affected by the type of nanoparticles or extracts used in the study. Notably, AuNPs showed low activity compared to AgNPs, possibly attributed to their characteristics and how they interact with bacterial cell membranes. Silver is well recognized for its properties, since it releases ions that disrupt cell membranes, influencing essential bacterial functions. Instead, gold may exhibit efficacy due to its unique chemical behavior and specific interactions with microorganisms. Therefore, the choice of the type of nanoparticles can significantly influence the efficiency of their antibacterial properties. Another mechanism displayed by AuNPs and AgNPs is based on inducing stress in the microbial cells, which triggers the formation of reactive oxygen species (ROS), with known disruptive effects. Among these effects, it is worth noticing the disintegration of the cell membrane, infiltration of exogenic components, diminished cellular respiratory function, and DNA impairment, culminating in cell demise [66].

Kocazorbaz et. al. [67] studied the effectiveness of *Q. coccifera* and AgNPs against *S. aureus, E. coli*, and *Pseudomonas aeruginosa*. The extract and AgNPs inhibited the growth of all strains at concentrations comparable to our research results [67]. However, *Q. infectoria* and AgNPs derived from this extract displayed effectiveness against *S. aureus, E. faecalis, Klebsiella pneumoniae*, and *Pseudomonas aeruginosa* [68]. Gold nanoparticles exhibited more effects than ethyl acetate extract from *Q. incana* on the tested pathogens, with inhibition zones increasing with higher concentrations [69]. Shalaby et. al. found that AgNPs were highly effective in inhibiting the bacteria *Enterococcus faecalis* and *Staphylococcus aureus*, surpassing the properties of kanamycin. Moreover, ZnONPs displayed effects against various Gram-negative bacteria, such as *Escherichia coli, Pseudomonas aeruginosa*, and *Salmonella typhimurium* [64].

### 3.7. Antifungal Activity

Among the tested samples, AgQP showed the strongest antifungal activity (Table 5). The controls used in this case were the initial nanoparticle synthesis agents, AgNO_3_ and HAuCl_4_. Ionic solutions at initial concentrations and microdilutions did not show antifungal activity. Moreover, the extracts used in the synthesis of AuNPs and AgNPs did not show antifungal activity for *Candida albicans* ATCC 10213 and for *Candida auris* ATCC 10913 at initial concentrations and microdilutions, except for *Candida krusei* ATCC 6258, where the MIC for QD and QF was 5 mg/mL and QP was 2.5 mg/mL. On the other hand, when the group of AuQD, AuQF, and AuQP is compared with the group of AgQD, AgQF, and AgQP, it can be noticed that AgNPs exhibit superior antifungal activity compared to AuNPs.

Gul et al. showed that the methanolic extract of *Q. infectoria* and the aqueous extract of *Q. infectoria* showed antifungal activity on *C. albicans* [70]. AgNPs produced through synthesis showed improved effectiveness when compared to fluconazole against *Phoma glomerata*, *Phoma herbarum*, *Fusarium semitectum*, *Trichoderma* sp., and *Candida albicans* [71]. The AgNPs produced naturally showed effectiveness against fungal pathogens responsible for different plant diseases, such as *Alternaria alternata, Sclerotinia sclerotiorum, Macrophomina phaseolina, Rhizoctonia solani, Botrytis cinerea*, and *Curvularia lunata,* when used at a dosage of 15 mg/mL [72]. Aljabali et. al. showed that AuNPs did not show antifungal properties, up to concentrations of 5 mg/mL, regardless of their size. The study suggests that the antimicrobial and antifungal effects are attributed not to the gold nanoparticles themselves but rather to the presence of ions that are responsible for the observed antibacterial and antifungal results [73]. In other research, it was revealed that AuNPs obtained using a *Tectona grandis* extract (at 6% concentration) and Au^3+^ (at a concentration of 2.9 × 10^3^ M) displayed antibacterial and antifungal properties. When compared to treatments, they exhibited effective activity against *Pseudomonas aeruginosa* (11 mm), *Aspergillus niger* (12 mm), *Bacillus subtilis* (12.5 mm), and *Escherichia coli* (15.5 mm) [74].

### 3.8. Cytotoxic Effects on Human Keratinocytes (HaCaT Cells)

**Figure 7:**
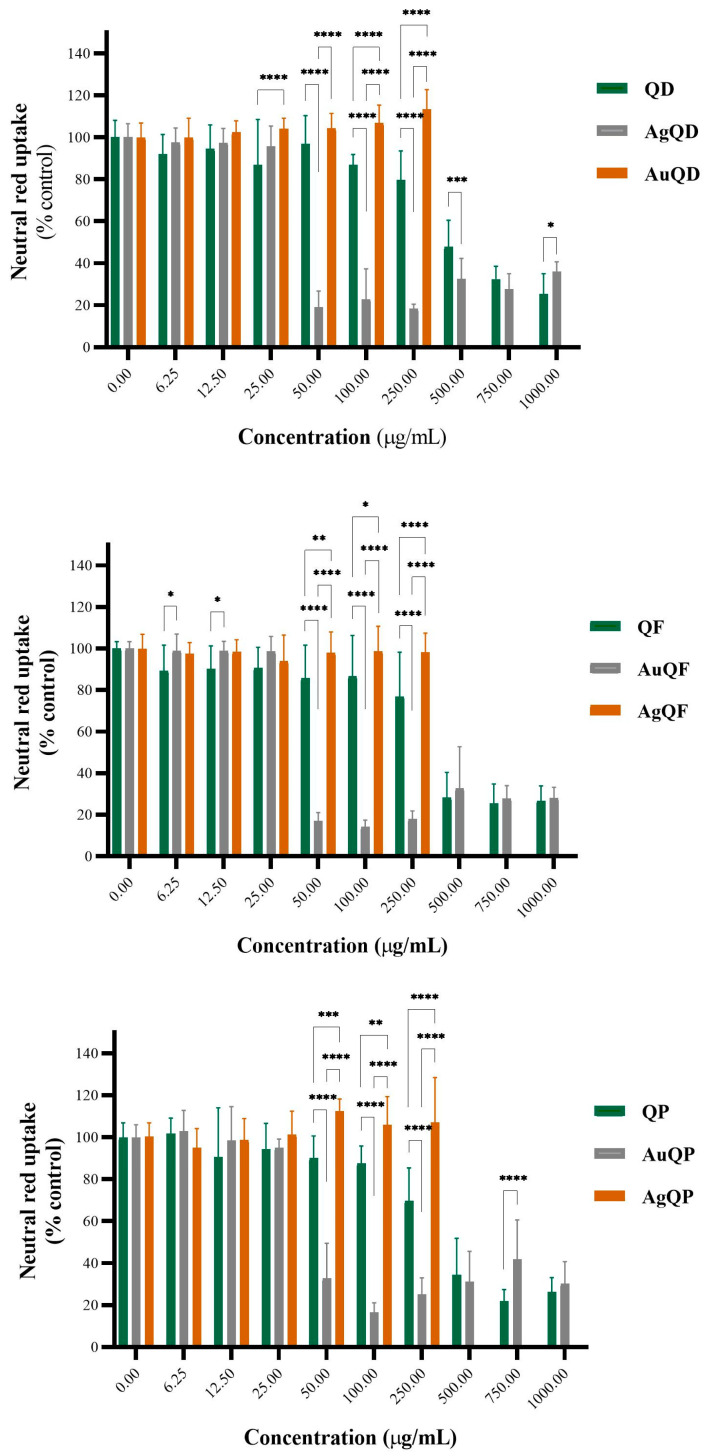
The cellular viability percentage of the HaCaT cell line determined by the NR assay after 24 h of incubation with various concentrations of AgNPs, AuNPs, and oak bark extracts. The results are presented as mean ± SD (n = 3). The NR assay showed a reduction in cellular viability greater than 50%, compared to the control. Data were considered to be statistically significant where * *p* < 0.05, ** *p* < 0.1, *** *p* < 0.01, and **** *p* < 0.001.

The cytotoxicity of several concentrations of AuQD, AuQF, AuQP, AgQD, AgQF, and AgQP and the QD, QF, and QP extracts was assessed 24 h after exposure using the neutral red (NR) uptake assay (Figure 7). In this study, it was demonstrated that 100 μg/mL of QD extract had no cytotoxic effect on the metabolic activity of HaCaT cells, while at concentrations higher than 250 μg/mL, up to 1000 μg/mL, it reduced the viability of HaCaT cells starting with 75%, up to 25% compared to the control. The same trend was observed for silver nanoparticles (AgNPs) obtained with that extract (AgQDs): up to 25 μg/mL, no significant effects on cell viability were recorded, but at concentrations higher than 50 μg/mL, a reduction of up to 70% in cell viability was found. As for the QF extract, it did not affect cell viability up to 250 μg/mL. However, at concentrations higher than 500 μg/mL, cellular viability decreased by 40% and, at concentrations of 750 μg/mL and 1000 μg/mL, decreased further to 70%. AgQF had a cytotoxic effect on HaCaT cells, reducing their viability by 80% at concentrations higher than 50 μg/mL. In the case of the QP extract, it did not cause significant alterations in cell viability at concentrations lower than 25 μg/mL. Conversely, at concentrations of 50 μg/mL and 100 μg/mL, it reduced cell viability by 80%, at 250 μg/mL by 65%, and at concentrations of 500 μg/mL, 750 μg/mL, and 1000 μg/mL it reduced viability by 82.5%. In the case of AgQP, at concentrations higher than 50 μg/mL, cell viability decreased by 70%. Accordingly, the studied extracts (QD, QF, and QP) and AgQD, AgQF, and AgQP significantly altered the viability of HaCaT cells at higher concentrations, while AuQD, AuQF, and AuQP did not show cytotoxic effects on HaCaT cell viability. Nevertheless, at the concentration of 1000 μg/mL, no significant changes were observed in the keratinocytes’ viability. These findings are presumably related to several relevant factors, including nanoparticles’ size, structure, and chemical properties. For example, differences in size and structure can impact the mechanistic interaction between nanoparticles and cell membranes, while chemical properties can influence their reactivity and toxic potential [75].

## 4. Spearman’s Correlations

**Figure 8:**
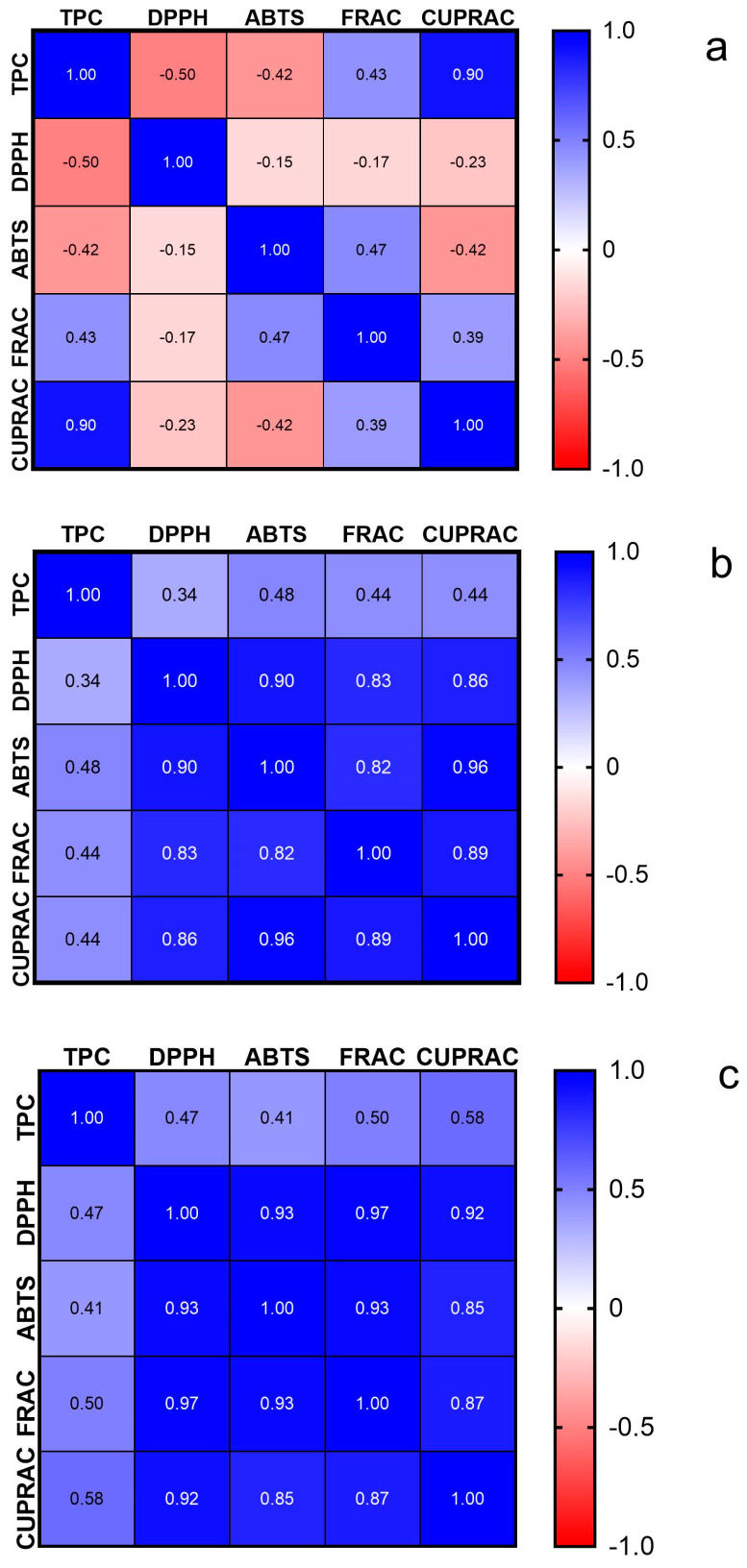
Spearman correlation coefficients for oak bark extracts (**a**), AuNPs (**b**), and AgNPs (**c**).

During statistical analysis, the Spearman correlation coefficient was helpful to showcase the relationship between TPC values and the in vitro antioxidant activity of the extracts (Figure 8a), AuNPs (Figure 8b), and AgNPs (Figure 8c).

The correlations were examined at significance levels of 1%, 5%, and 10%. For the extracts, a strong positive correlation (rho > 0.900) was found between TPC and CUPRAC (rho = 0.90, *p* = 0.002), while a weak and insignificant correlation was noted between TPC and FRAC (rho = 0.43, *p* = 0.2440). In terms of AuNPs, a weak correlation was observed between TPC and antioxidant effects according to Colton’s criteria. However, all four methods for evaluating antioxidant activity showed correlations among themselves with statistical significance. Between DPPH and ABTS, there is a correlation, as described by the values rho = 0.93 and *p* = 0.0011. The correlation between DPPH and CUPRAC is also notable at rho = 0.86 and *p* = 0.0029, while the association between DPPH and FRAC stands at rho = 0.83 with a *p* value of 0.0058. The relationship between ABTS and FRAC is relatively high, with rho = 0.82 and *p* = 0.0072, whereas the correlation between ABTS and CUPRAC is very strong, with rho = 0.96 and a significant *p*-value below 0.0001; finally, the link between FRAC and CUPRAC shows a correlation at rho = 0.89, with *p* = 0.0015. Similar correlations were noted for AgNPs as well. In summary, there was a statistically insignificant connection observed between TPC and the in vitro antioxidant activity; however, strong significant relationships were found among the individual DPPH, ABTS, FRAC, and CUPRAC values.

## 5. Conclusions

The obtained results suggest that *Quercus* bark extracts can be used as suitable reducing agents in the green synthesis of silver (AgNPs) and gold (AuNPs) nanoparticles, with the botanical identity of the species affecting the chemical and bioactive properties of the obtained nanoparticles.

Future studies will focus on delving into how these nanoparticles in particular interact with cell membranes and other biological components, highlighting the processes through which they negatively influence bacterial and fungal cells, while being beneficial for human cells. The objective is to assess the impacts of nanoparticles on cell varieties and their lasting effects on living organisms, encompassing biodistribution, metabolism, and removal investigations. These findings are driving the exploration of uses for nanoparticles, encompassing their application in antimicrobial treatments, medical substances, and drug distribution systems. The ultimate objective is to translate this research into applications by conducting trials and establishing guidelines for medical use.

## Figures and Tables

**Table 1:** Phenolic compounds identified and quantified in QD, QF, QP, AuQD, AuQF, AuQP, AgQD, AgQF, AgQP, expressed in µg/mL, detected at 260 nm.

Compound	RT (min)	QD	QF	QP	AuQD	AuQF	AuQP	AgQD	AgQF	AgQP
Gallic acid	2.96	14.81	9.37	40.79	0.09	NF	0.01	NF	NF	NF
Eleutheroside B	4.89	13.98	65.74	1.50	NF	NF	NF	NF	NF	NF
Chlorogenic acid	4.94	3.19	1.37	0.64	0.01	NF	0.02	0.02	0.02	NF
Catechin	4.97	30.02	12.38	1.50	0.02	NF	0.04	0.03	NF	NF
Epicatechin	5.76	72.17	161.54	36.99	0.24	0.01	NF	0.38	0.43	0.31
Luteolin-3′,7-di-O—glucoside	5.90	8.99	6.38	2.04	NF	NF	NF	NF	NF	NF
Caffeic acid	6.01	12.63	19.81	NF	0.29	NF	NF	NF	NF	NF
Vanillic acid	6.04	10.16	NF	5.18	NF	NF	NF	NF	NF	0.07
Luteolin-7-O-glucoside	6.89	130.62	71.08	NF	NF	NF	NF	NF	NF	NF
Ellagic acid	7.18	1477.26	8077.98	4902.84	14.88	NF	NF	NF	NF	NF
Sinapic acid	7.95	3.13	NF	0.71	NF	NF	NF	NF	NF	NF
Taxifolin	8.05	4.90	16.06	0.11	0.02	0.02	0.01	0.19	0.01	NF
Quercetin	10.63	123.90	21.20	14.65	NF	NF	NF	8.24	NF	NF
Total (μg/mL)		1905.76	8462.91	5006.94	15.557	0.03	0.084	8.864	0.466	0.381

Note: NF—not found or concentration below the limit of detection. QD—*Q. dalechampii*, QF—*Q. frainetto*, QP—*Q. petraea,* AuQD—gold nanoparticles obtained via *Q. dalechampii* rhytidome, AuQF—gold nanoparticles obtained via *Q. frainetto* rhytidome, AuQP—gold nanoparticles obtained via *Q. petraea* rhytidome, AgQD—silver nanoparticles obtained via *Q. dalechampii* rhytidome, AgQF—silver nanoparticles obtained via *Q. frainetto* rhytidome and AgQP—silver nanoparticles obtained via *Q. petraea* rhytidome. Standard calibration curves: Eleutheroside B (*y* = 16,834.13*x* + 31,559.97; *R*^2^ = 0.992); Catechin (*y* = 16,753.88*x* + 6711.08; *R*^2^ = 0.999); Chlorogenic acid (*y* = 46,063.75*x* + 76,630.16 < *R*^2^ = 0.984); Epicatechin (*y* = 7927.63*x* + 6711.08; *R*^2^ = 0.979); Caffeic acid (*y* = 20,210.25*x* + 20,982.75; *R*^2^ = 0.993); Vanillic acid (*y* = 45,252.80*x* + 88,403.66; *R*^2^ = 0.991); Luteolin-7-*O*-glucoside (*y* = 36,800.23*x* + 132,152.29; *R*^2^ = 0.995); Rutin (*y* = 23,287.83*x* + 48,225.26; *R*^2^ = 0.997); Ellagic acid (*y* = 8348.39*x* + 724,935.97; *R*^2^ = 0.998); Taxifolin (*y* = 27,268.68*x* + 13,259.25; *R*^2^ = 0.998); Sinapic acid (*y* = 74,425.41*x* + 47,024.62; *R*^2^ = 0.998); Quercetin (*y* = 36,077.91*x* + 53,183.83; *R*^2^ = 0.998).

**Table 2:** Selected FT-IR bands according to chemical groups identified during the spectral analysis of *Quercus* extracts and derived nanoparticles.

Wavenumber (cm^−1^)	Chemical Vibration
3500–3200	OH stretching
2931–2937	Asymmetric and symmetric vibrations of C-H-, CH_2_-, and CH_3_- from polysaccharides
1710–1716	C=O stretching
1603–1605	Primary amine
1506–1520	C=C aromatic symmetrical stretching
1445–1447	C-C stretch from aromatics compounds
1336–1342	Aromatic nitro compounds
1196–1232	C-O stretching vibration
1033–1049	C-C and C-H ring vibration of cyclic molecules and C-OH stretching

**Table 3:** Overview of TPC and antioxidant capacity values measured for AuNP, AgNP, and *Quercus* extracts.

	TPC (mgGAE/g dw)	DPPH (mgTE/g dw)	ABTS (mgTE/g dw)	FRAP (mgTE/g dw)	CUPRAC (mgTE/g dw)
QD	407.0 (386.2–413.5) ^c^	2050 ± 24.74 ^f^	129.38 ± 136.45 ^e^	2350.31 ± 21.33 ^g^	810.69 ± 15.91 ^c^
AgQD	192.6 (183.3–192.6) ^a^	302.97 ± 10.56 ^d^	446.38 ± 4.25 ^c^	332.94 ± 3.88 ^d^	332.98 ± 2.19 ^a^
AuQD	132.7 (132.2–133.8) ^a^	119.01 ± 3.11 ^b^	129.38 ± 5.32 ^b^	138.22 ± 1.96 ^b^	287.10 ± 2.63 ^a^
QF	437.9 (424.7–450.5) ^c^	1424 ± 32.49 ^g^	67.27 ± 170.76 ^d^	1578.17 ± 19.25 ^h^	844.89 ± 7.43 ^c^
AgQF	196.7 (191.1–201.6) ^a^	220.89 ± 18.64 ^c^	426.38 ± 7.23 ^c^	306.96 ± 1.24 ^d^	318.35 ± 2.74 ^a^
AuQF	53.06 (52.44–53.06) ^b^	72.25 ± 19.73 ^b^	67.27 ± 1.72 ^b^	82.17 ± 1.77 ^c^	102.15 ± 2.54 ^b^
QP	319.8 (318.2–337.0) ^a^	972.9 ± 16.40 ^h^	114.10 ± 86.57 ^f^	1025.88 ± 7.22 ^f^	665.59 ± 7.47 ^a^
AgQP	290.4 (277.2–295.5) ^a^	446.34 ± 2.54 ^e^	515.88 ± 9.10 ^c^	552.30 ± 12.15 ^e^	474.77 ± 13.22 ^a^
AuQP	47.14 (46.53–48.36) ^b^	114.79 ± 1.28 ^b^	114.10 ± 2.05 ^b^	122.33 ± 0.88 ^b^	138.07 ± 0.62 ^b^

Data for TPC are shown as median and interquartile range (Q1–Q3), and for DPPH, ABTS, FRAP, CUPRAC are shown as mean ± standard deviation. Statistical analysis was performed by Kruskal–Wallis (Dunn’s post-test) for TPC, and for the antioxidant activity it was performed with One-way Anova (Turkey’s post-test). n = 9, and values followed by different superscript letters (a–g) in the same column are significantly different. DPPH, free radical scavenging activity against 2,2-diphenyl-1-picrylyl radicals; TE, Trolox equivalents; ABTS, free radical scavenging activity against 2,2′-azino-bis(3-ethylbenzothiazoline-6-sulfonate); TE, Trolox equivalents; FRAP, ferric reducing antioxidant power; Fe(II) chelating capacity, ferrous ion chelating capacity; CUPRAC, cupric reducing antioxidant capacity.

**Table 4:** Antibacterial activity, expressed as MIC/MBC (mg/mL), of AuNPs, AgNPs, and oak bark extracts, against selected bacteria strains.

	*S. aureus* ATCC 25923	MRSA ATCC 43300	*E. faecalis* ATCC 29212	*E. coli* ATCC 25922	*K. pneumoniae* ATCC 13883	*P. aeruginosa* ATCC 27853
QD	0.62/2.50	0.62/>5.00	>5.00/>5.00	>5.00/>5.00	0.62/>5.00	2.50/5.00
AgQD	1.25/2.50	1.25/2.50	2.50/>5.00	2.50/2.50	1.25/1.25	1.25/5.00
AuQD	2.50/>5.00	2.50/>5.00	>5.00/>5.00	>5.00/>5.00	2.50/2.50	>5.00/>5.00
QF	0.62/>5.00	0.62/>5.00	>5.00/>5.00	>5.00/>5.00	0.62/>5.00	1.25/5.00
AgQF	0.62/2.50	1.25/1.25	2.50/2.50	2.50/2.50	1.25/1.25	1.25/2.50
AuQF	1.25/>5.00	2.50/2.50	>5.00/>5.00	>5.00/>5.00	2.50/2.50	>5.00/>5.00
QP	1.25/5.00	0.31/>5.00	>5.00/>5.00	>5.00/>5.00	0.31/0.62	0.62/2.50
AgQP	0.31/1.25	1.25/2.50	1.25/2.50	1.25/1.25	1.25/1.25	0.62/1.25
AuQP	2.50/>5.00	5.00/5.00	>5.00/>5.00	>5.00/>5.00	2.50/2.50	>5.00/>5.00

Note: QD—*Q. dalechampii*, QF—*Q. frainetto*, QP—*Q. petraea,* AuQD—gold nanoparticles obtained via *Q. dalechampii* rhytidome, AuQF—gold nanoparticles obtained via *Q. frainetto* rhytidome, AuQP—gold nanoparticles obtained via *Q. petraea* rhytidome, AgQD—silver nanoparticles obtained via *Q. dalechampii* rhytidome, AgQF—silver nanoparticles obtained via *Q. frainetto* rhytidome and AgQP—silver nanoparticles obtained via *Q. petraea* rhytidome.

**Table 5:** Evaluation of antifungal activity, expressed as MIC/FMC (mg/mL), of AuNPs, AgNPs, and oak bark extracts.

	*Candida albicans* ATCC 10213	*Candida krusei* ATCC 6258	*Candida auris* ATCC 10913
QD	>5.00/>5.00	5.00/>5.00	>5.00/>5.00
AgQD	1.25/>5.00	0.04/0.63	1.25/>5.00
AuQD	>5.00/>5.00	5.00/>5.00	5.00/>5.00
QF	>5.00/>5.00	5.00/>5.00	>5.00/>5.00
AgQF	1.25/5.00	0.02/0.16	1.25/>5.00
AuQF	>5.00/>5.00	5.00/>5.00	5.00/>5.00
QP	>5.00/>5.00	2.50/>5.00	>5.00/>5.00
AgQP	0.62/2.50	0.01/0.16	0.63/2.50
AuQP	>5.00/>5.00	1.25/>5.00	5.00/>5.00

## Data Availability

Data is contained within the article.
